# Deep learning system for malignancy risk prediction in cystic renal lesions: a multicenter study

**DOI:** 10.1186/s13244-024-01700-0

**Published:** 2024-05-20

**Authors:** Quan-Hao He, Jia-Jun Feng, Ling-Cheng Wu, Yun Wang, Xuan Zhang, Qing Jiang, Qi-Yuan Zeng, Si-Wen Yin, Wei-Yang He, Fa-Jin Lv, Ming-Zhao Xiao

**Affiliations:** 1 Department of Urology, The First Affiliated Hospital of Chongqing Medical University, Chongqing, People’s Republic of China; 2 Department of Medical Imaging, Guangzhou First People’s Hospital, School of Medicine, South China University of Technology, Guangzhou, China; 3 Department of Urology, Sun Yat-Sen Memorial Hospital, Sun Yat-Sen University, Guangzhou, People’s Republic of China; 4 Department of Urology, Yongchuan Hospital of Chongqing Medical University, Chongqing, China; 5 Department of Urology, The Second Affiliated Hospital of Chongqing Medical University, Chongqing, China; 6 Department of Urology, Chongqing University Fuling Hospital, Chongqing, People’s Republic of China; 7 Department of Radiology, The First Affiliated Hospital of Chongqing Medical University, Chongqing, People’s Republic of China

**Keywords:** Cystic renal lesions, Bosniak-2019 classification, Deep learning, Radiomics

## Abstract

**Objectives:**

To develop an interactive, non-invasive artificial intelligence (AI) system for malignancy risk prediction in cystic renal lesions (CRLs).

**Methods:**

In this retrospective, multicenter diagnostic study, we evaluated 715 patients. An interactive geodesic-based 3D segmentation model was created for CRLs segmentation. A CRLs classification model was developed using spatial encoder temporal decoder (SETD) architecture. The classification model combines a 3D-ResNet50 network for extracting spatial features and a gated recurrent unit (GRU) network for decoding temporal features from multi-phase CT images. We assessed the segmentation model using sensitivity (SEN), specificity (SPE), intersection over union (IOU), and dice similarity (Dice) metrics. The classification model’s performance was evaluated using the area under the receiver operator characteristic curve (AUC), accuracy score (ACC), and decision curve analysis (DCA).

**Results:**

From 2012 to 2023, we included 477 CRLs (median age, 57 [IQR: 48–65]; 173 men) in the training cohort, 226 CRLs (median age, 60 [IQR: 52–69]; 77 men) in the validation cohort, and 239 CRLs (median age, 59 [IQR: 53–69]; 95 men) in the testing cohort (external validation cohort 1, cohort 2, and cohort 3). The segmentation model and SETD classifier exhibited excellent performance in both validation (AUC = 0.973, ACC = 0.916, Dice = 0.847, IOU = 0.743, SEN = 0.840, SPE = 1.000) and testing datasets (AUC = 0.998, ACC = 0.988, Dice = 0.861, IOU = 0.762, SEN = 0.876, SPE = 1.000).

**Conclusion:**

The AI system demonstrated excellent benign-malignant discriminatory ability across both validation and testing datasets and illustrated improved clinical decision-making utility.

**Critical relevance statement:**

In this era when incidental CRLs are prevalent, this interactive, non-invasive AI system will facilitate accurate diagnosis of CRLs, reducing excessive follow-up and overtreatment.

**Key Points:**

- The rising prevalence of CRLs necessitates better malignancy prediction strategies.
- The AI system demonstrated excellent diagnostic performance in identifying malignant CRL.
- The AI system illustrated improved clinical decision-making utility.

**Graphical Abstract:**

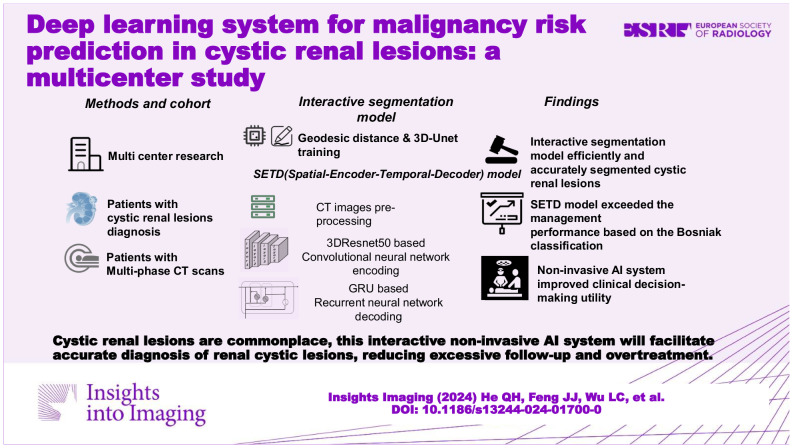

## Introduction

Cystic renal lesions (CRLs) are being detected more frequently as CT becomes more commonplace [1, 2]. While a small number of CRLs are malignant renal neoplasms requiring surgical intervention, most CRLs are benign cystic renal neoplasms or simple renal cysts which do not require surgery [3, 4]. Precisely identifying the components of CRLs is crucial for deciding on suitable treatment approaches. Contrast-enhanced computed tomography (CECT) imaging is frequently utilized to distinguish among the different types of CRLs [5]. However, the complex pattern of malignant CRLs on CECT images, including septal thickness, mural nodule enhancement, and calcifications presence, pose significant challenges for both diagnosis and management, particularly when dealing with early-stage cases [6].

Existing limitations in diagnosing malignant CRLs necessitate novel approaches [2]. While the Bosniak-2019 classification aimed to improve accuracy, recent studies have shown limited improvement compared to its predecessor [3]. Additionally, the updated criteria reclassify a significant portion of previously diagnosed class III lesions, which also adds to the follow-up burden. The misapplication of Bosniak categorization can lead to inaccurate treatment and associated diagnostic errors, resulting in adverse outcomes such as impairment of renal function, re-operation surgery, and increased medical disputes [7].

To overcome the challenges of subjective biases in visual image evaluations and improve diagnostic sensitivity (SEN), machine learning algorithms, trained on quantitative data extracted from CT scans, are demonstrating potential in differentiating between benign and malignant CRLs [8]. Minisk et al identified six features from nephrogenic CT phase scans, while Dana et al focused on extracting radiomics features from nephrogenic CT phase scans. Both studies achieved promising results in differentiating between benign and malignant CRLs by modeling these features [4, 9].

Our prior study has developed a blending ensemble machine learning algorithm integrating quantitative radiomics features with deep learning features, which demonstrated favorable classification performance in accurately differentiating between benign and malignant CRLs [10]. However, the prior study only utilized corticomedullary phase CT images to build the machine learning algorithm instead of multi-phase CT scans. In CECT phases imaging (renal protocol), renal mass enhancement is assessed by analyzing the difference in Hounsfield units (HU) before and after contrast administration. An increase of fifteen or more HU value within the solid tumor regions indicates enhancement, highlighting critical malignant renal neoplasms components [11]. In this study, we aim to develop and validate an interactive 3D U-Net segmentation model which can efficiently segment CRLs in corticomedullary and nephrogenic phase CT images [12]. To enhance the SEN and specificity (SPE) of diagnosing CRLs, a spatial encoder temporal decoder (SETD) deep learning classifier was created and validated. SETD model utilizes a pre-trained 3D ResNet50 encoder block to extract spatial features and a gated recurrent unit (GRU) network to decode temporal features from multi-phase CT scans.

## Methods

### Study participants and inclusion criteria

Since this study was retrospective, informed consent was not required, and the research ethics committees of each participating hospital approved it. This study is in accordance with the CLEAR checklist guidelines [13]. In the segmentation model cohort, the inclusion criteria were as follows: individuals with CRLs comprising less than 25% solid tissue, diameter more than 1 cm, and absence of history of prior kidney surgery or polycystic kidney disease.

**Fig. 1:**
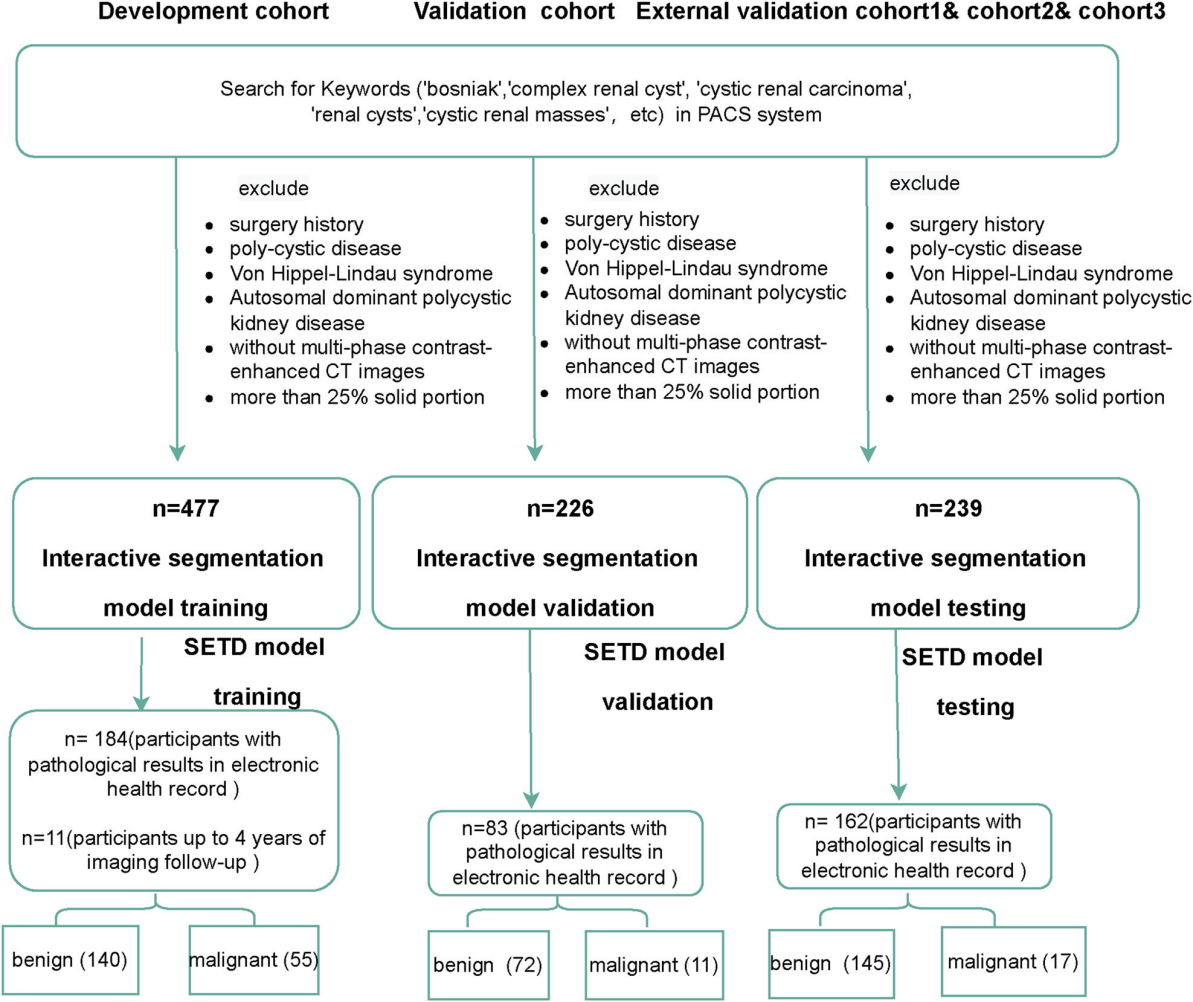
Flowchart of the enrollment process and pathology results for cystic renal lesions (CRLs) in each cohort. In the training cohort, benign CRLs were identified using pathology criteria and a 4-year CT imaging follow-up. The validation cohort was employed for model validation. The external validation cohort 1, cohort 2, and cohort 3 were combined for model testing

To ensure the generation of an optimal machine learning model applicable to clinical practice, the inclusion criteria for the SETD model’s training cohort were histopathological analysis results after surgery as well as CRLs cases with no progression observed in CT or MRI imaging over a period of four years. To guarantee the reliability of the SETD model’s performance, all enrolled participants in the SETD validation and testing datasets had confirmed histological diagnosis results. During the construction of the SETD model, CRLs were incorporated in a balanced distribution across the Bosniak classification categories, enabling the model to capture the full variability of imaging appearances. Detailed inclusion and exclusion criteria are displayed in Fig. 1.

### CT acquisition protocol

The training cohort (The First Affiliated Hospital of Chongqing Medical University), validation cohort (The Second Affiliated Hospital of Chongqing Medical University), external validation cohort 1 (Guangzhou First People’s Hospital), and external validation cohort 3 (Chongqing University Fuling Hospital) used 128-slice or 64-slice spiral CT scanners to obtain contrast-enhanced CT scans. External validation cohort 2 (Yongchuan Hospital of Chongqing Medical University) used a 256-slice spiral CT scanner. All cohorts used standardized CT imaging scanning protocols (Appendix Table E2). The prior study only utilized corticomedullary phase CT images to build the machine learning algorithm [10]. In this study, the corticomedullary phase and nephrogenic phase CT images were utilized to create the artificial intelligence (AI) system.

### ROI sketching and Bosniak-2019 system reclassification quality control

All CRLs regions of interest (ROIs) labeling and Bosniak-2019 reclassification was undertaken by two abdominal radiologists (with more than 10 years clinical experience in diagnostic radiology). To precisely delineate the CRL margin, radiologists carefully reviewed multi-phase CT image data using ITK-SNAP software, incorporating information from the corticomedullary and nephrogenic phases across axial, sagittal, and coronal planes. In cases where there was contentious CRL sketching and conflicting classifications, another senior radiologist (FJ-L, over 20 years of hands-on clinical experience in diagnostic radiology, particularly in the interpretation of multi-phase CT scans) would participate in the discussion. This collaboration ensured a consensus was reached on the final ROIs sketching and Bosniak-2019 reclassification outcomes.

### Interactive segmentation model architecture

The proposed deep interactive segmentation method based on 3D U-Net and geodesic distance transforms is depicted in Appendixes E1 and E2. To reduce the interactions required with radiologists, we implemented a two-stage 3D U-Net segmentation approach, consisting of an initial Proposal Network (P-net) for segmentation, followed by a Refinement Network (R-net) for enhancing the segmentation accuracy. P-net provides an initial automatic segmentation by using a raw image with single channel. The radiologist then verifies the segmentation results and adds some interactions (clicks or scribbles) to indicate the mis-segmented regions and 3D boundaries of target CRLs. R-net takes the original image, the initial segmentation, and the user interactions (encompassing a total of four image channels) as inputs to provide a refined segmentation result. This iterative process allows the radiologist to repeatedly provide feedback to R-net, refining the outcomes based on P-net’s preliminary automatic segmentation results.

### Spatial encoder and temporal decoder model architecture

**Fig. 2:**
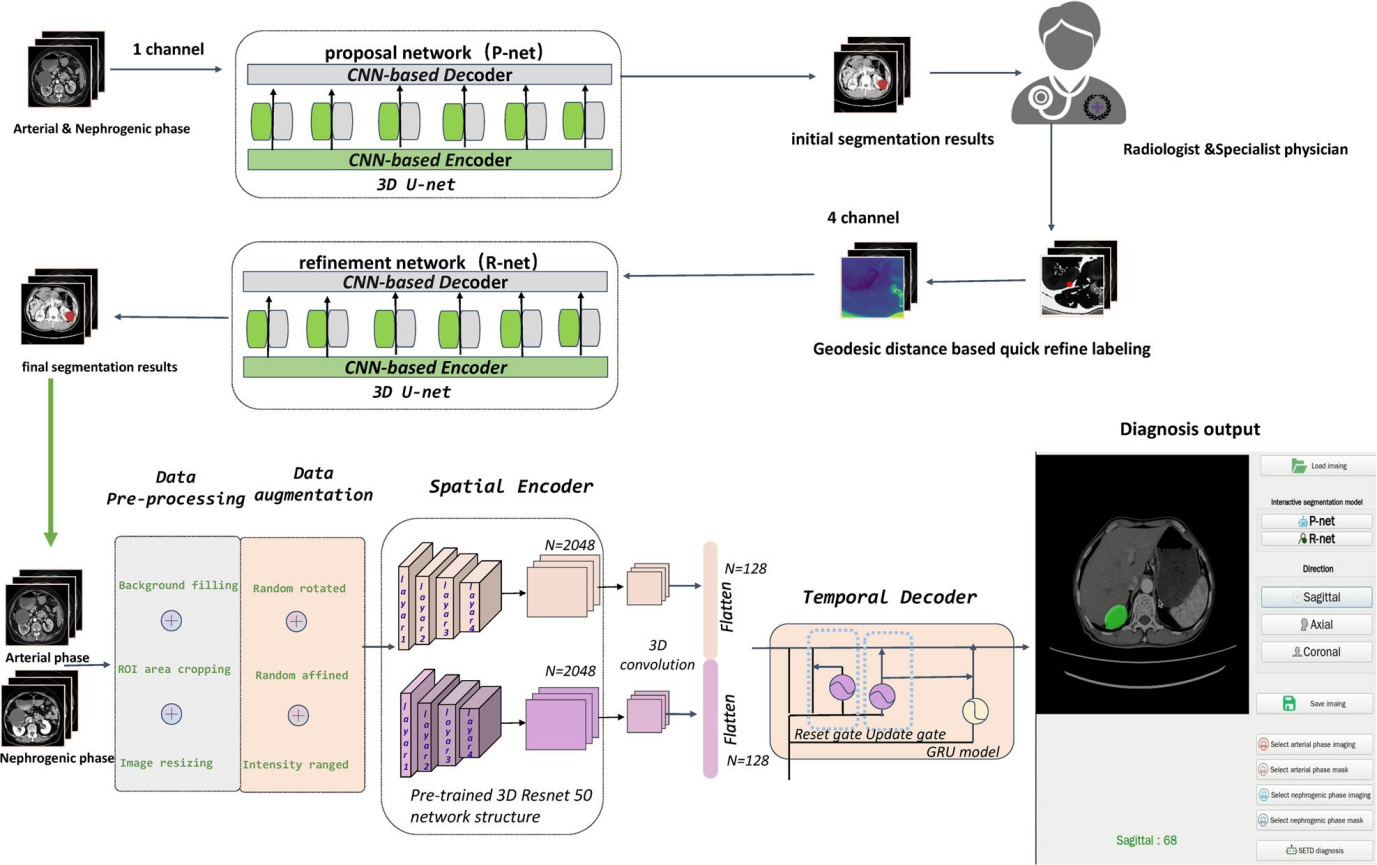
Flowchart providing a detailed procedures used to construct the CRLs artificial intelligence (AI) system

The SETD model adopts an encoder-decoder based modular design, which takes preprocessed multiphase CT images as input and generates the corresponding CRL malignancy probability. To extract spatial features from multi-phase CT images, the spatial encoder module uses a deep convolutional neural network composed of the encoder layers of 3DResnet50 network with pre-trained weights. Following the outputs of the spatial encoder module, the SETD incorporates a temporal decoder module. This module consists of two GRU layers. The GRU network is particularly effective in identifying evolving patterns across the multiphase CT images, a critical aspect in the differential diagnosis of malignant renal tumors. The final step in the process involves passing the GRU output through a linear layer, which calculates the probability of malignancy (Fig. 2).

### Statistical analysis

SEN, SPE, Intersection over union (IOU), and dice similarity (Dice) were used to evaluate the segmentation model. The area under the receiver operator characteristic curve (AUC), decision curve analysis (DCA), and accuracy score (ACC) were utilized to evaluate the performance of SETD model compared with prior machine learning models and Bosniak-2019 version. The level of statistical significance was determined by two-sided p value of less than 0.05. All models were implemented using MONAI framework (version 1.12, https://monai.io/). The interactive segmentation model, SETD model and data processing procedure are publicly available on GitHub (https://github.com/jacobhqh1997/CRL_AI).

## Results

### Patient clinical characteristics

The characteristic distributions of the patients in each cohort are summarized in Table 1. From 2012 to 2023, we included 477 CRLs in the training cohort, 226 CRLs in the validation cohort, and 239 CRLs in the testing cohort (external validation cohort 1, cohort 2, and cohort 3).

**Table 1:** Baseline characteristics of patients with CRLs

	Training cohort	Validation cohort	External validation cohort 1	External validation cohort 2	External validation cohort 3
Patients (*n*)	403	150	31	44	87
Age (median-IQR)	57 (48–65)	60 (52–68.75)	58 (53–63.50)	60.50 (53–70)	60 (51.5–70.5)
Sex (male)	173 (42.9%)	77 (51.3%)	17 (54.8%)	29 (65.9%)	49 (56.3%)
Sex (female)	230 (57.1%)	73 (48.7%)	14 (45.2%)	15 (34.1%)	38 (43.7%)
Interactive segmentation model					
Total renal cysts	477	226	44	74	121
Patients with isolated cysts	355	119	21	27	66
Patients with ipsilateral cysts	11	8	1	0	7
Patients with contralateral cysts	25	6	8	11	10
Patients with ipsilateral and contralateral cysts	12	17	1	6	5
SETD model					
CRLs diameter (mean-SD, cm)	6.53 ± 2.99	6.61 ± 2.04	6.52 ± 2.51	6.55 ± 2.11	6.27 ± 1.96
Up to 4 years of imaging follow-up (benign)	11	0	0	0	0
Benign (*n* = 346)	129	72	26	36	83
Malignant (*n* = 83)	55	11	5	8	4

In the SETD training cohort, all imaging follow-up assessments of CRLs were conducted over a duration of up to 4 years. All CRLs in the SETD external validation cohorts had histopathological results

*CRLs* cystic renal lesions, *SETD* spatial encoder temporal decoder

Based on the 2005 Bosniak version, 11 CRLs in the SETD training cohort were originally categorized as Bosniak II (8 lesions) and III (3 lesions). After a 4-year imaging follow-up, these were confirmed to be benign renal cysts. Additionally, one CRL was reclassified to Bosniak IV according to the updated 2019 Bosniak criteria. Histopathological results have been obtained for all lesions within the SETD validation and test cohorts, along with malignant CRLs in the SETD training cohort.

### Interactive segmentation model performance

**Fig. 3:**
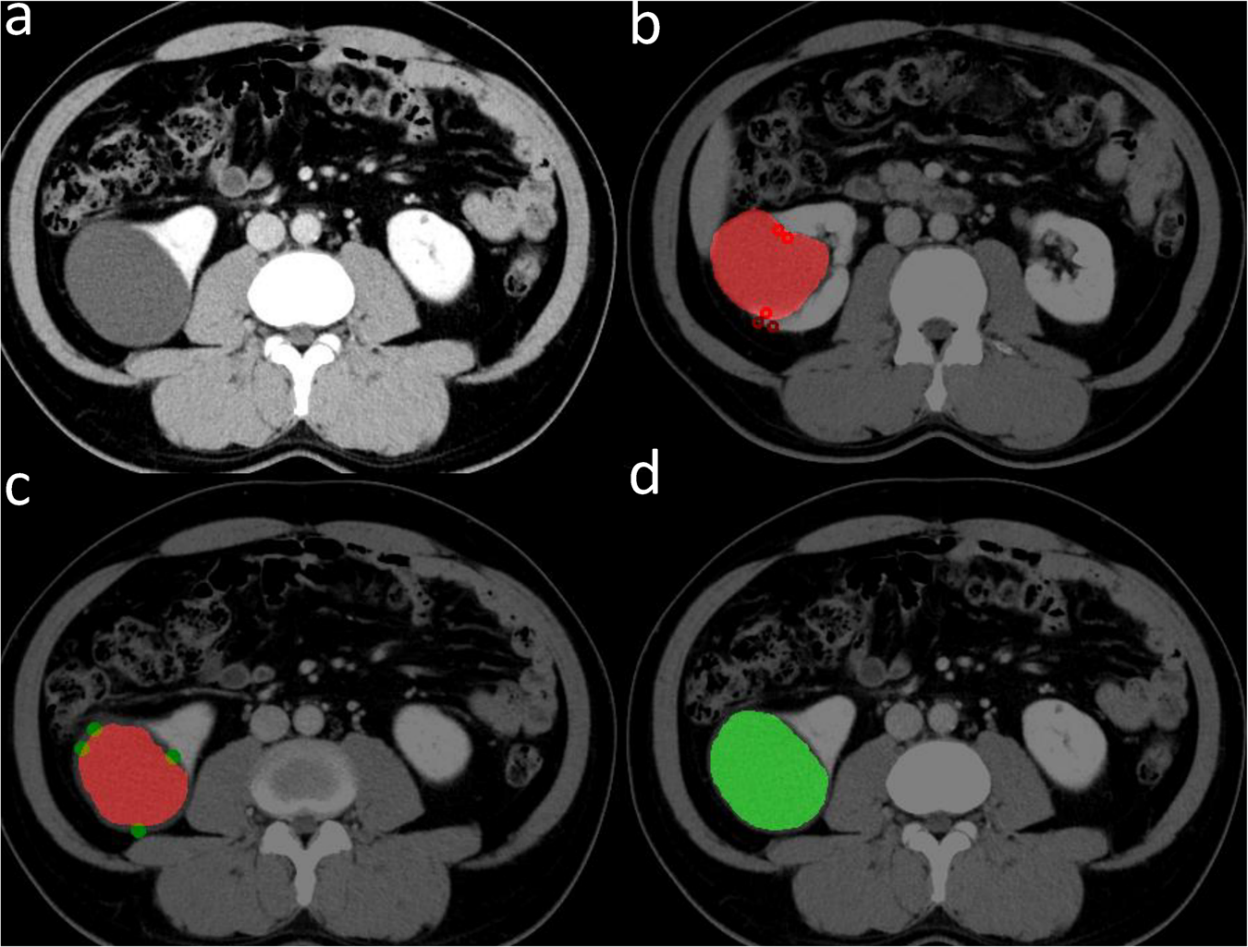
Example usage of the initial CRLs segmentation model and refined CRLs segmentation model. **a** Original image inputs, (**b**) initial segmentation results combined with negative indicator points (red dots), (**c**) initial segmentation results combined with positive indicator points (green dots), (**d**) refined segmentation results

Detailed performance metrics for both P-net and R-net in the validation and testing datasets are presented in Table 2. The proposed 3D R-net exhibited superior accuracy (validation: Dice = 0.847, IOU = 0.743, SEN = 0.840, SPE = 1.000; testing: Dice = 0.861, IOU = 0.762, SEN = 0.876, SPE = 1.000) with geodesic distance compared to the P-net (validation: Dice = 0.781, IOU = 0.713, SEN = 0.840, SPE = 1.000; testing: Dice = 0.803, IOU = 0.735, SEN = 0.775, SPE = 1.000). Figure 3 shows an example usage of the initial CRLs segmentation and refined CRLs segmentation by using radiologist-provided margin points, respectively.

**Table 2:** Performance of P-net and R-net models in validation and testing datasets

model	Dice (%)	IOU (%)	SEN (%)	SPE (%)
P-net (validation cohort)	78.10 ± 30.15	71.29 ± 29.55	84.01 ± 10.15	99.98 ± 0.04
P-net (test cohort)	80.30 ± 27.53	73.52 ± 28.39	77.45 ± 29.43	99.99 ± 0.02
R-net (validation cohort)	84.66 ± 8.63	74.26 ± 11.48	84.01 ± 10.15	99.98 ± 0.04
R-net (test cohort)	86.09 ± 7.15	76.23 ± 10.30	87.64 ± 8.58	99.96 ± 0.08

All performance metrics are reported as mean values along with their respective standard deviations

*Dice* dice similarity, *SEN* sensitivity, *SPE* specificity

### SETD classifier performance in CRL classification

The SETD model was evaluated on the validation and testing datasets using the best training weights. The model achieved a SEN of 100% and a SPE of 90.3% for the malignancy prediction in the validation cohort, and a SEN of 100% and a SPE of 97.2% for the malignancy prediction in the testing cohort.

### Bosniak-2019 system performance in CRL classification

The Bosniak-2019 classification system classified CRLs into two groups: benign (I, II) and potentially malignant (IIF, III, IV). The system achieved a SEN of 100% and a SPE of 68.1% for the malignancy prediction in the validation cohort, and a SEN of 88.2% and a SPE of 85.5% for the malignancy prediction in the testing cohort.

### Machine learning algorithm performance in CRL classification

We extensively assessed the preceding four machine learning algorithms based on our earlier research. The detailed radiomic features used in the previous machine learning algorithms are described in the Appendix E4. The Blending ensemble classifier was the best-performing model in the validation set (AUC = 0.902 (95% CI: 0.768–1.000), ACC = 0.940 (95% CI: 0.938–0.941)). The lightgbm classifier was the best-performing model in the testing set (AUC = 0.968 (95% CI: 0.942–0.993), ACC = 0.883 (95% CI: 0.881–0.884)). The detailed performance metrics of the four models are presented in Table 3.

**Table 3:** Performance of each model and Bosniak-2019 classification in validation and testing datasets

	Model	AUC (95% CI)	ACC (95% CI)	Sensitivity	Specificity	*p* Value
Validation cohort	SETD	0.973 (0.944–1.000)	0.916 (0.914–0.917)	100 (11/11)	90.3 (65/72)	*p* < 0.01
	Blending	0.902 (0.768–1.000)	0.940 (0.938–0.941)	90.9 (10/11)	91.7 (66/72)	*p* = 0.40
	Lightgbm	0.891 (0.774–1.000)	0.928 (0.926–0.929)	90.9 (10/11)	77.8 (56/72)	*p* *=* 0.43
	Decision tree	0.783 (0.662–0.904)	0.687 (0.682–0.692)	90.9 (10/11)	65.3 (47/72)	*p* *=* 0.41
	XGBoost	0.897 (0.769–1.000)	0.904 (0.902–0.906)	81.8 (9/11)	87.5 (63/72)	*p* *=* 0.42
	Bosniak 2019 classification	0.840 (0.786–0.895)	0.723 (0.718–0.728)	100 (11/11)	68.1 (49/72)	Reference
Test cohort	SETD	0.998 (0.993–1.000)	0.988 (0.988–0.988)	100 (17/17)	97.2 (141/145)	*p* < 0.01
	Blending	0.951 (0.910–0.993)	0.889 (0.888–0.890)	76.5 (13/17)	93.8 (136/145)	*p* *=* 0.09
	Lightgbm	0.968 (0.942–0.993)	0.883 (0.881–0.884)	94.1 (16/17)	64.8 (94/145)	*p* *=* 0.02
	Decision tree	0.902 (0.857–0.946)	0.840 (0.838–0.841)	100 (17/17)	64.1 (93/145)	*p* *=* 0.49
	XGBoost	0.939 (0.882–0.996)	0.883 (0.881–0.884)	94.1 (16/17)	90.3 (131/145)	*p* *=* 0.17
	Bosniak 2019 classification	0.869 (0.785–0.953)	0.858 (0.857–0.859)	88.2 (15/17)	85.5 (124/145)	Reference

*AUC* area under the receiver operating characteristic curve, *ACC* accuracy score, *p value* *p* value in DeLong test, *95% CI* 95% confidence interval

### Clinical utility of SETD classifier

**Fig. 4:**
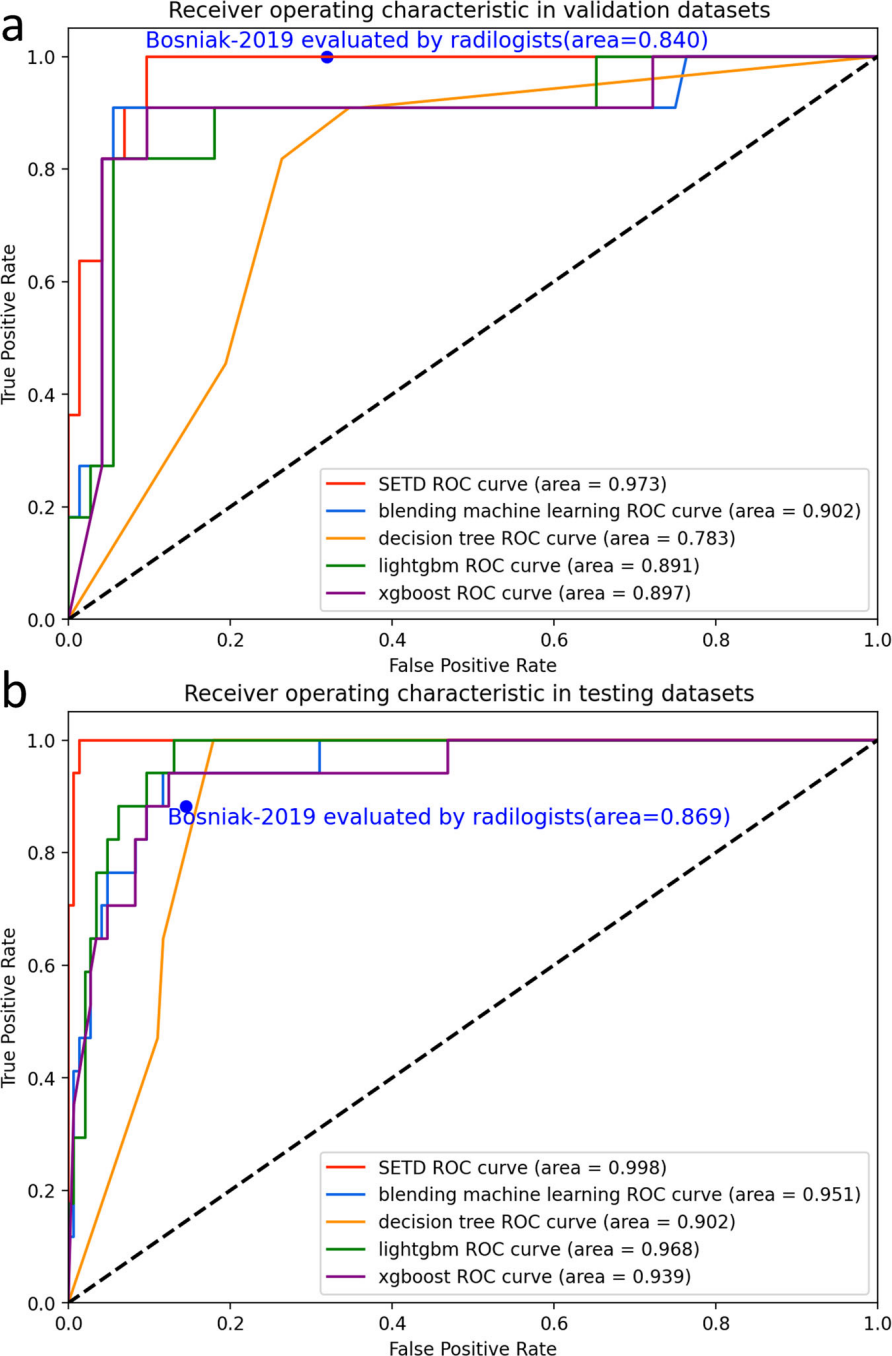
The diagnostic performance of each model evaluated by receiver operating characteristic (ROC) curve. **a** ROC curve in validation dataset, (**b**) ROC curve in testing dataset

**Fig. 5:**
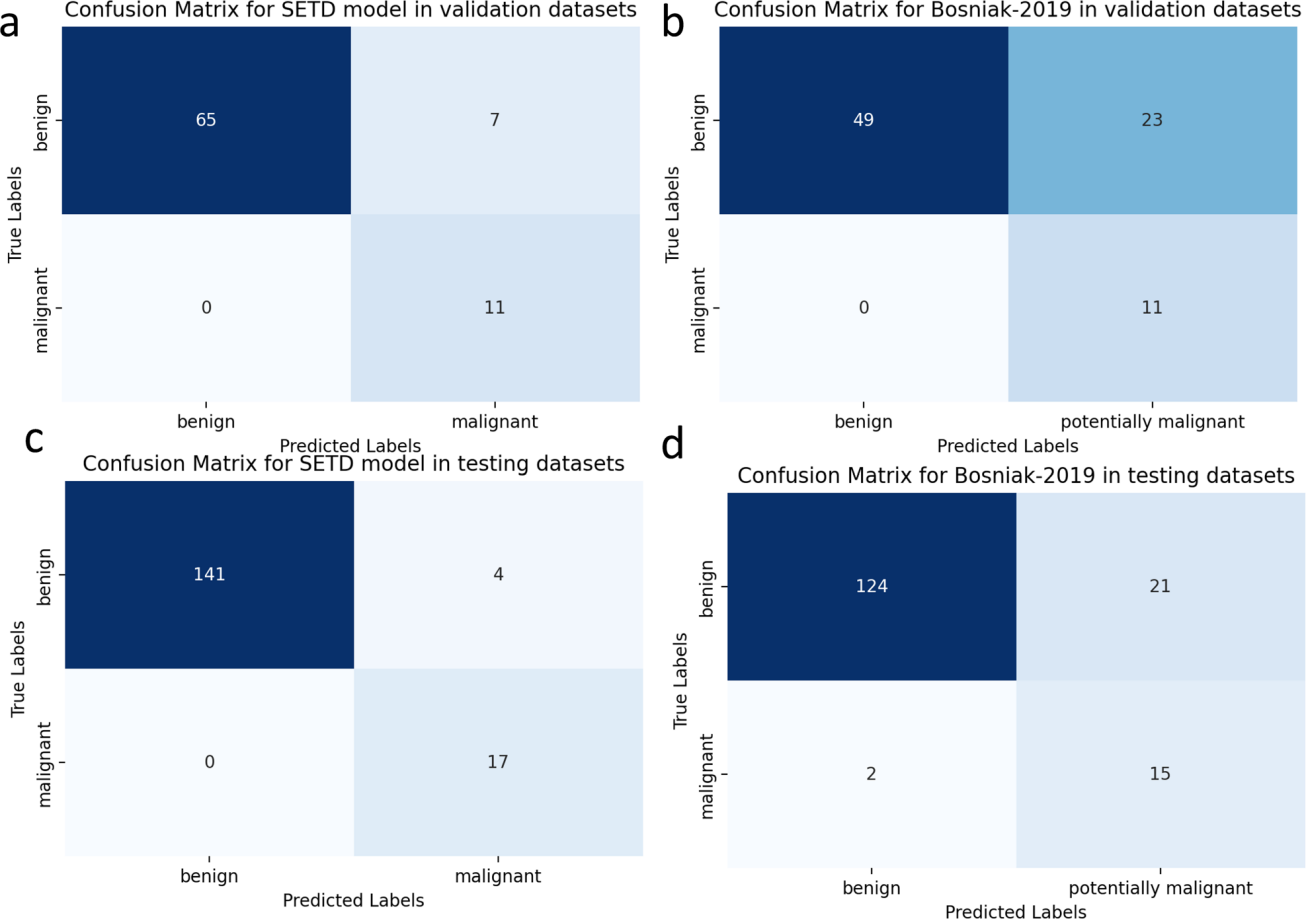
Confusion matrices for the SETD Model and the Bosniak-2019 classification. **a** Confusion matrix for SETD model in validation datasets, (**b**) confusion matrix for Bosniak-2019 in validation datasets, (**c**) confusion matrix for SETD model in testing datasets, (**d**) confusion matrix for Bosniak-2019 in testing datasets

Figure 4 presents a comparative analysis of the AUC performance of each model alongside the Bosniak-2019 classification, with the latter being specifically evaluated by radiologists. Figure 5 shows a detailed comparison between the confusion matrix of the SETD model and the Bosniak-2019 classification, with the Bosniak classification being evaluated by radiologists.

**Fig. 6:**
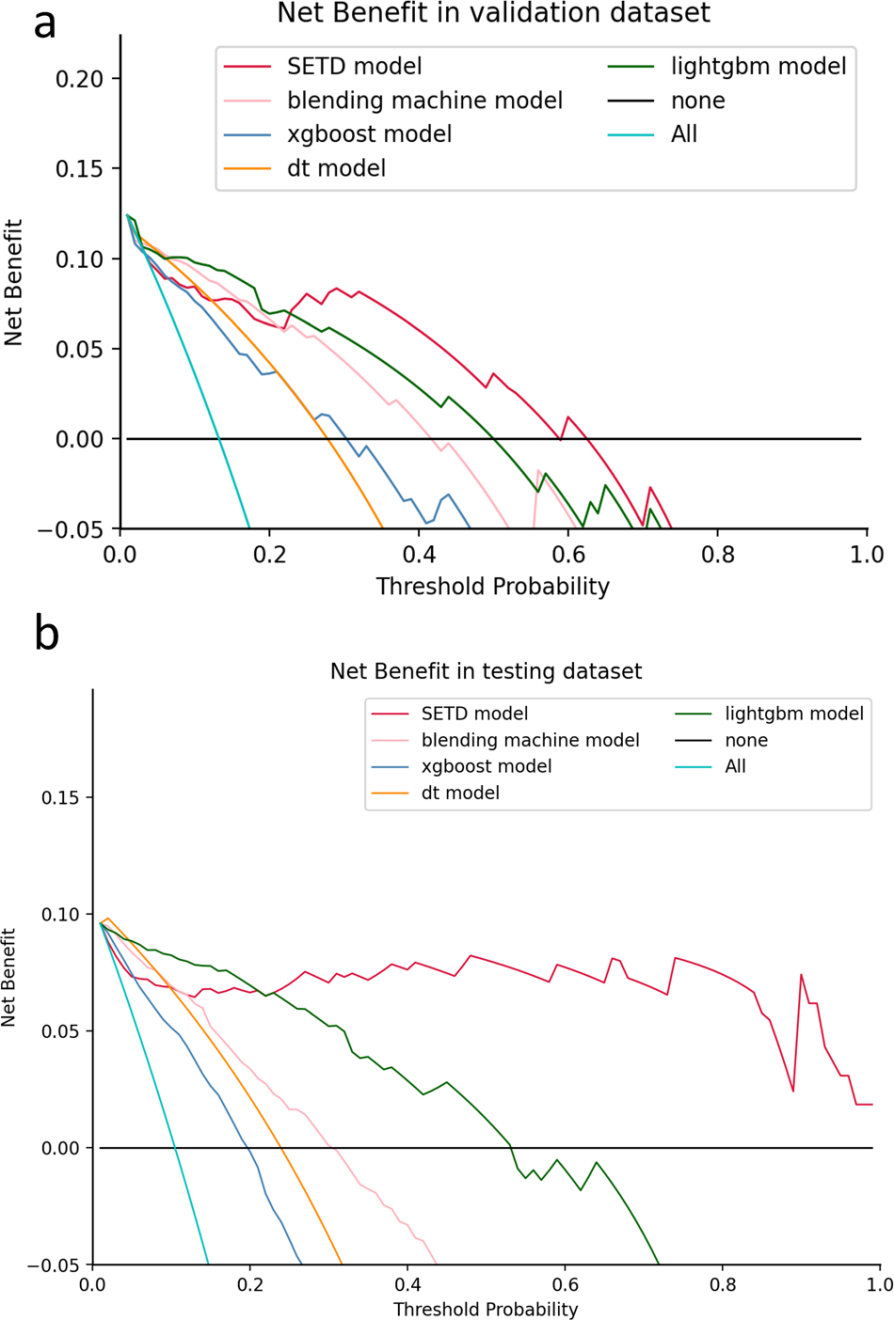
Decision Curve Analysis (DCA) for each model in validation and testing dataset. **a** DCA in validation dataset, (**b**) DCA in testing dataset. The *y*-axis represents the net benefit, while the *x*-axis represents the corresponding threshold probability. The SETD classifier is represented by red line

In the validation and testing datasets, the SETD model demonstrated a statistically significant higher AUC value (validation: 0.973, 95% CI: 0.944–1.000; testing: 0.998, 95% CI: 0.993–1.000) compared to the Bosniak-2019 classification, as indicated by the AUC DeLong test (*p* < 0.05). Figure 6 depicts the results of DCA in each model performed on validation and testing datasets, showing that the SETD model provides a greater net benefit than the “none” and “all” treatment strategies for all considered threshold probabilities. The deep learning system outperformed the Bosniak-2019 based management guideline in both the validation and testing datasets, suggesting that clinical decision support could be improved by employing the SETD algorithm. The Appendix Figs. E3 and E4 shows a detailed confusion matrix for machine learning models and the Bosniak-2019 classification.

## Discussion

In this retrospective multicenter study, we developed a non-invasive AI system to stratify malignant and benign CRLs. The reliability and applicability of the AI algorithm are determined by the following key factors: 1. Radiologists can provide feedback to the interactive CRL segmentation model, which can improve the segmentation results and make the process more efficient and accurate. 2. Histopathologic results serve as the diagnostic gold standard for CRL classification in both validation and testing datasets. 3. Early stopping approaches and weighted cross-entropy loss prevented overfitting in SETD training steps. 4. The SETD classifier demonstrated strong diagnostic performance in both validation and testing datasets. 5. The calculated RQS quality score of this study is 43, which demonstrates that the AI algorithm construction is trustworthy and repeatable.

In diagnosing malignant renal neoplasms, evaluating changes in HU value before and after CECT scans is highly effective. For example, clear cell renal cell carcinoma (RCC) typically exhibits a fast-in, fast-out CT presentation in CECT scans. Chromophobe RCC and Papillary RCC tend to be relatively homogeneous contrast enhancement in  CECT scans [14]. During the corticomedullary and nephrographic phases, benign CRLs, such as simple renal cysts and cystic nephroma, typically exhibit a more gradual and less pronounced intense enhancement [15]. We developed the SETD model to detect useful features from multi-phase CT images, with a focus on feature changes in multi-phase CT images. This greatly improved the model’s predictive performance and interpretability [16]. Given that feature-based machine learning algorithms are more sensitive to variations in imaging parameters, the SETD algorithm exhibits a more robust performance in external validation dataset compared to the machine learning model used in our previous study.

A growing body of evidence suggests that cystic RCC is less aggressive and has a better prognosis than solid RCC of the same size [17]. One plausible explanation for this is that only the solid portions of malignant CRLs contain malignant cells, which generally make up a very small portion of the lesion’s overall volume [18]. Developing reliable methods to differentiate benign cysts from malignant tumors, especially in larger cysts is crucial. Risk stratification based on our deep learning system can help guide management decisions. Low-risk cysts may be monitored conservatively or treated with minimally invasive procedures like laparoscopic decortication, while high-risk cysts may require more extensive evaluation or intervention [19].

Updated guidelines from various medical organizations have shifted toward more conservative approaches to manage renal cysts [20]. Active surveillance has become the recommended option for Bosniak IIF and even some Bosniak III and IV lesions since the majority of CRLs are benign renal cysts [21, 22]. The updated Bosniak classification version aims to reduce inter-reader variability and enhance the accuracy of malignancy predictions in CRLs. However, the effectiveness of this revised classification in clinical practice still requires validation. Moreover, the implications of prolonged medical follow-up for high-level Bosniak renal cysts should be carefully considered. To our knowledge, this is the largest CRLs related AI research with multicenter validation, providing a more reliable tool for preoperatively discriminating malignant CRLs. A recent study on IIF-classified CRLs with a median follow-up duration of 50 months showed a significantly lower incidence of IIF renal lesions upgrading and malignancy than previously reported. The research also observed that all participants did not demonstrate any significant radiological progression beyond 36 months of follow-up. This indicates that follow-up beyond 36 months may not be necessary in most cases [23]. In this research, the SETD model delivered a clear, concise, and binary (yes/no) determination regarding the likelihood of malignancy in CRLs. This demonstrates its practical utility in clinical settings, aiding radiologists and urologists in evaluating and selecting the optimal surgical strategy for CRL treatment.

Our research has several limitations. Firstly, its retrospective design necessitates further prospective validation to reinforce findings. Secondly, the strict inclusion and exclusion criteria may introduce selection bias, suggesting the need for broader international multi-center studies to confirm the generalizability of our results. Thirdly, all ROIs in interactive segmentation model training were labeled by radiologists, and this approach seems a little bit old-fashioned despite it can produce more reliable and convincing results. Our follow-up study will examine whether semi-supervised segmentation methods can be applied in clinical practice to reduce radiologists’ workload [24]. Lastly, clinical characteristics like age and gender may be potential predictors. A mixture model that combines deep learning networks with clinical features may further improve the diagnostic model’s performance.

In conclusion, we developed and validated a novel AI system, combining an interactive 3D U-Net segmentation model with a SETD model, to accurately differentiate between benign and malignant CRLs. The AI algorithm demonstrated good discrimination capability across validation and testing datasets. The non-invasive AI system will facilitate accurate diagnosis of renal cystic lesions, reducing excessive follow-up and overtreatment.

### Supplementary information

Electronic Supplementary Material

Supplementary Movie E1

## Data Availability

The datasets analyzed during the current study are not publicly available due to the need for follow-up research but are available from the corresponding author on reasonable request.

## Abbreviations

ACC: Accuracy score
AUC: Area under the receiver operator characteristic curve
CI: Confidence interval
CRLs: Cystic renal lesions
DCA: Decision curve analysis
Dice: Dice similarity
GRU: Gated recurrent unit
IOU: Intersection over union
ROI: Regions of interest
RCC: Renal cell carcinoma
SETD: Spatial encoder temporal decoder
