# Supplementary material for: Deep learning system for malignancy risk prediction in cystic renal lesions: a multicenter study
Source: Insights Imaging. 2024 May 20;15:121. doi: 10.1186/s13244-024-01700-0 (PMC11102892; doi:10.1186/s13244-024-01700-0)
Supplement: Supplementary file 1 — Electronic Supplementary Material [file 13244_2024_1700_MOESM1_ESM.pdf]

Deep learning system for malignancy risk prediction in cystic renal lesions: a multicenter study

ELECTRONIC SUPPLEMENTARY MATERIAL

Appendix E1. Detailed structure of the 3D U-Net model

Appendix E2. Geodesic transformation example in CT images

Appendix E3. Detailed SETD loss function explanation

Appendix E4. Radiomic features adopted in machine learning model

Appendix E5. CRLs Bosniak-2019 version reclassification procedure

Appendix E6. Detailed structure components and parameters in SETD model

Appendix E7. Deep learning model training process

Appendix E8. AI-assisted diagnosis case example

Figure E1. Detailed workflow of CT images preprocessing for SETD model training

Figure E2. Detailed workflow of CT images augmentation for SETD model training

Figure E3. The confusion matrix for the machine learning model in validation datasets

Figure E4. The confusion matrix for the machine learning model in testing datasets

Figure E5. The calibration curve for the SETD model and machine learning model

Table E1. Deep learning and radiomics model quality score checklist

Table E2. Detailed CT imaging scanning protocols in the training cohort and each validation cohort

Table E3. Detailed distribution of Bosniak-2019 classification and pathology results in the SETD model training cohort and external validation cohorts.

CLEAR Checklist

Movie E1 legend. Example usage of the cystic renal lesions AI diagnosis system

Appendix E1. Detailed structure of the 3D U-Net model

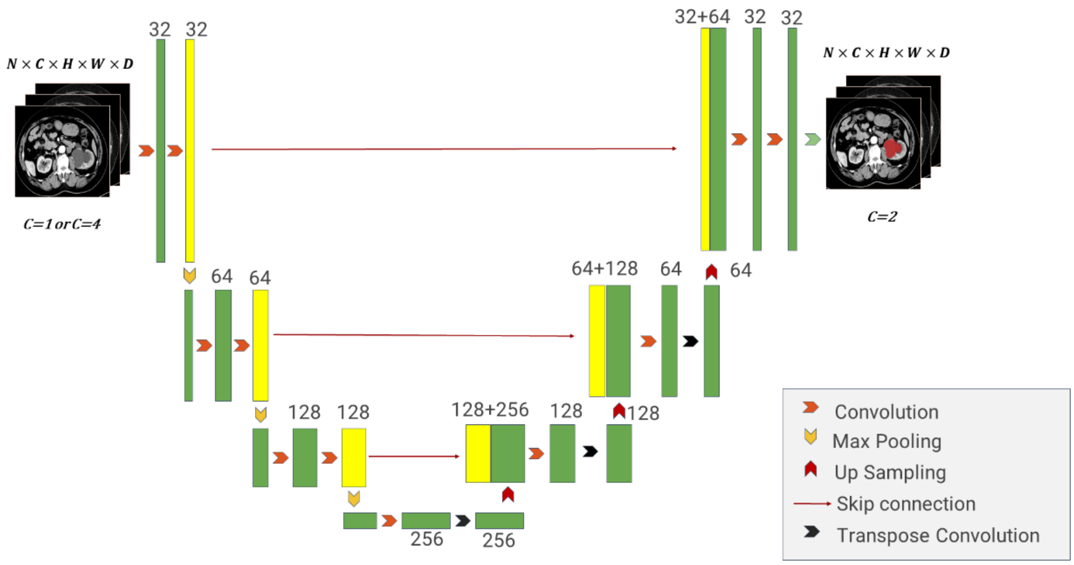

The 3D U-Net network is designed for the segmentation task of cystic renal lesions (CRLs)[1]. The proposal segmentation network (P-net) and the refinement segmentation network (R-net) have the same 3D U-Net network structure. The P-net input has one channel and R-net input has four channels (the original image, the P-net initial segmentation results and the user interactions)[2; 3].

Appendix E2. Geodesic transformation example in CT images

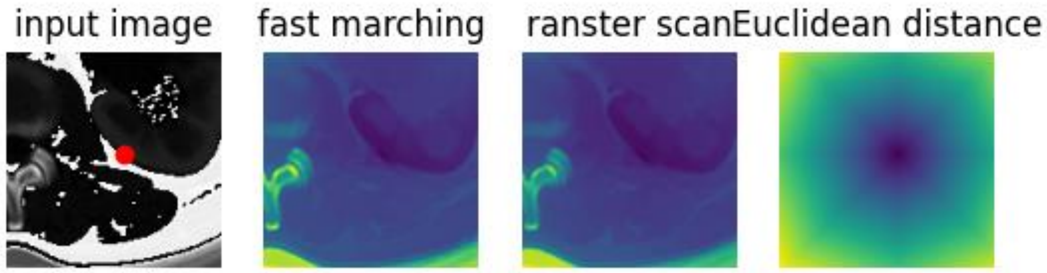

Geodesic transformation is a mathematical procedure employed for the smoothing and segmentation of medical images. It operates by determining the shortest path between each pixel in the image and a designated seed point. Pixels closer to the seed point receive higher values, represented by a color gradient (red point above), while those farther away are assigned lower values. Unlike Euclidean distance transformation, the geodesic transformation approaches such as fast marching and raster scan can take into account the intensity variation of the image, which makes them more accurate for segmenting medical objects with complex boundaries[4; 5].

Appendix E3. Detailed SETD loss function explanation

Considering the sample imbalance in the training set, we adopted a cross-entropy loss function with sample weights. The details are as follows:

1. Calculation of class frequencies

Calculate the frequency of occurrence of each class c in the training set. N represents the total number of training samples, count_c is the number of occurrences of class c in the training set.

$$\text{freqS}_C = \frac{\text{count}_c}{N}$$

2. Calculation of the class weights

$$\text{class\_weightS}_C = \frac{1}{\log(1.02 + \text{freqS}_C)}$$

3. Definition of cross-entropy loss function with sample weights

Define the cross-entropy loss function with sample weights. c is the number of classes, y_c is a binary indicator (0 or 1) whether the sample belongs to class c, p_c is the predicted probability of the sample belonging to class c, and w_c is the weight assigned to class c.

$$\text{CELoss} = -\sum_{c=1}^{c} w_c y_c \log(p_c)$$

4. Batch-wise loss computation during training

During training, compute the loss for a single batch size. B is the batch size, C is the number of classes, $y_{b,c}$ is a binary indicator (0 or 1) whether the b-th sample in the batch belongs to class C, $p_{b,c}$ is the predicted probability of the b-th sample in the batch belonging to class C, and w_c is the weight assigned to class C.

$$\text{Loss} = -\frac{1}{B}\sum_{b=1}^{B}\sum_{c=1}^{C} w_c y_{b,c} \log(p_{b,c})$$

This approach aims to alleviate the impact of imbalanced class distributions by adjusting the significance of each class through sample weights. The objective is to minimize this weighted loss over the entire training dataset, facilitating the refinement of model parameters to enhance its ability to accurately predict probabilities for imbalanced classes within a batch[6; 7].

Appendix E4. Radiomic features adopted in machine learning model

Gray-Level Co-Occurrence Matrix (GLCM) features

$$\text{Informational measure of correlation 2 (IMC2)} = \sqrt{1 - e^{-2(HXY2 - HXY)}}$$

$$\text{cluster shade} = \sum_{i=1}^{N_g}\sum_{j=1}^{N_g} (i + j - u_x - u_y)^3 p(i, j)$$

$$\text{Informational Measure of Correlation 1 Imc1} = \frac{HXY - HXY1}{\max\{HX, HY\}}$$

Gray Level Size Zone Matrix (GLSZM) Features

$$\text{SmallAreaEmphasis} = \frac{\sum_{i=1}^{N_g}\sum_{j=1}^{N_s}\frac{P(i_j)}{j^2}}{N_z}$$

$$\text{ZoneVariance} = \sum_{i=1}^{Ng}\sum_{j=1}^{N_s} p(i, j)(j - u)^2$$

$$\text{GrayLevelNonUniformity} = \frac{\sum_{i=1}^{N_g}\left(\sum_{j=1}^{N_r} P(i, j|\theta)\right)^2}{N_r(\theta)}$$

$$\text{SizeZoneNonUniformityNormalized} = \frac{\sum_{j=1}^{N_s}\left(\sum_{i=1}^{N_q} P(i, j)\right)^2}{N_z^2}$$

Gray Level Run Length Matrix (GLRLM) Features

$$\text{LongRunLowGrayLevelEmphasis} = \frac{\sum_{i=1}^{N_g} \sum_{j=1}^{N_r} \frac{P(i,j|\theta)j^2}{i^2}}{N_r(\theta)}$$

$$\text{RunEntropy} = -\sum_{i=1}^{Ng} \sum_{j=1}^{N_r} p(i,j|\theta)\log_2 (p(i,j|\theta) + \epsilon)$$

Shape Features (3D)

$$\text{flatness} = \sqrt{\frac{\lambda_{least}}{\lambda_{major}}}$$

Gray Level Dependence Matrix (GLDM) Features

$$\text{SmallDependenceHighGrayLevelEmphasis} = \frac{\sum_{i=1}^{N_q} \sum_{j=1}^{N_d} \frac{P(i,j)}{i^2 j^2}}{N_z}$$

$$\text{Dependence Entropy} = -\sum_{i=1}^{Ng} \sum_{j=1}^{N_d} p(i,j)\log_2 (p(i,j) + \epsilon)$$

First Order Features

$$\text{Mean} = \frac{1}{N_P}\sum_{i=1}^{Np} x(i)$$

Median =The median intensity value of X

Image Types

Wavelet: Wavelet filtering yields 8 decompositions per level (all possible combinations of applying either a high or a low pass filter in each of the three dimensions).

LoG: Laplacian of Gaussian filter, edge enhancement filter. Emphasizes areas of gray level change, where sigma defines how coarse the emphasized texture should be. A low sigma emphasis on fine textures (change over a short distance), where a high sigma value emphasizes coarse textures (gray level change over a large distance).

Original: No filter applied

The online documentation for Pyradiomics describes and provides the precise calculation methods for hand-crafted radiomic characteristics (https://pyradiomics.readthedocs.io/en/latest/features.html).

We start features extraction by using the standard sample parameters settings provided in the official Pyradiomics YAML file, and CT images are resampled to 1×1×1 mm^3 voxels to standardize the slice thickness. Image intensities will be binned by 25 HU, and voxel array shift is set to 1000. All radiomic features that we extract adhere to the feature criteria set by the Imaging Biomarker Standardization Initiative[8].

Appendix E5. CRLs Bosniak-2019 version reclassification procedure

Two professional abdominal radiologists independently reclassify CRLs in the training, validation, and testing datasets according to the Bosniak-2019 version. They are blind to the corresponding pathological results. In the case of a contentious CRL classification, another senior radiologist (FJ-L, over 20 years of hands-on clinical experience in diagnostic radiology) will participate in the discussion and help develop the final decision together.

Appendix E6. Detailed structure components and parameters in SETD model

SETD model structure			
Conv3d-1	Conv3d-91	ReLU-181	ReLU-271
BatchNorm3d-2	BatchNorm3d-92	MaxPool3d-182	Conv3d-272
ReLU-3	ReLU-93	Conv3d-183	BatchNorm3d-273
MaxPool3d-4	Conv3d-94	BatchNorm3d-184	ReLU-274
Conv3d-5	BatchNorm3d-95	ReLU-185	Conv3d-275
BatchNorm3d-6	ReLU-96	Conv3d-186	BatchNorm3d-276
ReLU-7	Conv3d-97	BatchNorm3d-187	ReLU-277
Conv3d-8	BatchNorm3d-98	ReLU-188	Bottleneck-278
BatchNorm3d-9	ReLU-99	Conv3d-189	Conv3d-279
ReLU-10	Bottleneck-100	BatchNorm3d-190	BatchNorm3d-280
Conv3d-11	Conv3d-101	Conv3d-191	ReLU-281
BatchNorm3d-12	BatchNorm3d-102	BatchNorm3d-192	Conv3d-282
Conv3d-13	ReLU-103	ReLU-193	BatchNorm3d-283
BatchNorm3d-14	Conv3d-104	Bottleneck-194	ReLU-284
ReLU-15	BatchNorm3d-105	Conv3d-195	Conv3d-285
Bottleneck-16	ReLU-106	BatchNorm3d-196	BatchNorm3d-286
Conv3d-17	Conv3d-107	ReLU-197	ReLU-287
BatchNorm3d-18	BatchNorm3d-108	Conv3d-198	Bottleneck-288
ReLU-19	ReLU-109	BatchNorm3d-199	Conv3d-289
Conv3d-20	Bottleneck-110	ReLU-200	BatchNorm3d-290
BatchNorm3d-21	Conv3d-111	Conv3d-201	ReLU-291
ReLU-22	BatchNorm3d-112	BatchNorm3d-202	Conv3d-292
Conv3d-23	ReLU-113	ReLU-203	BatchNorm3d-293
BatchNorm3d-24	Conv3d-114	Bottleneck-204	ReLU-294
ReLU-25	BatchNorm3d-115	Conv3d-205	Conv3d-295
Bottleneck-26	ReLU-116	BatchNorm3d-206	BatchNorm3d-296
Conv3d-27	Conv3d-117	ReLU-207	ReLU-297
BatchNorm3d-28	BatchNorm3d-118	Conv3d-208	Bottleneck-298
ReLU-29	ReLU-119	BatchNorm3d-209	Conv3d-299
Conv3d-30	Bottleneck-120	ReLU-210	BatchNorm3d-300
BatchNorm3d-31	Conv3d-121	Conv3d-211	ReLU-301
ReLU-32	BatchNorm3d-122	BatchNorm3d-212	Conv3d-302
Conv3d-33	ReLU-123	ReLU-213	BatchNorm3d-303
BatchNorm3d-34	Conv3d-124	Bottleneck-214	ReLU-304
ReLU-35	BatchNorm3d-125	Conv3d-215	Conv3d-305
Bottleneck-36	ReLU-126	BatchNorm3d-216	BatchNorm3d-306
Conv3d-37	Conv3d-127	ReLU-217	ReLU-307
BatchNorm3d-38	BatchNorm3d-128	Conv3d-218	Bottleneck-308
ReLU-39	ReLU-129	BatchNorm3d-219	Conv3d-309

Conv3d-40	Bottleneck-130	ReLU-220	BatchNorm3d-310
BatchNorm3d-41	Conv3d-131	Conv3d-221	ReLU-311
ReLU-42	BatchNorm3d-132	BatchNorm3d-222	Conv3d-312
Conv3d-43	ReLU-133	Conv3d-223	BatchNorm3d-313
BatchNorm3d-44	Conv3d-134	BatchNorm3d-224	ReLU-314
Conv3d-45	BatchNorm3d-135	ReLU-225	Conv3d-315
BatchNorm3d-46	ReLU-136	Bottleneck-226	BatchNorm3d-316
ReLU-47	Conv3d-137	Conv3d-227	ReLU-317
Bottleneck-48	BatchNorm3d-138	BatchNorm3d-228	Bottleneck-318
Conv3d-49	ReLU-139	ReLU-229	Conv3d-319
BatchNorm3d-50	Bottleneck-140	Conv3d-230	BatchNorm3d-320
ReLU-51	Conv3d-141	BatchNorm3d-231	ReLU-321
Conv3d-52	BatchNorm3d-142	ReLU-232	Conv3d-322
BatchNorm3d-53	ReLU-143	Conv3d-233	BatchNorm3d-323
ReLU-54	Conv3d-144	BatchNorm3d-234	ReLU-324
Conv3d-55	BatchNorm3d-145	ReLU-235	Conv3d-325
BatchNorm3d-56	ReLU-146	Bottleneck-236	BatchNorm3d-326
ReLU-57	Conv3d-147	Conv3d-237	Conv3d-327
Bottleneck-58	BatchNorm3d-148	BatchNorm3d-238	BatchNorm3d-328
Conv3d-59	Conv3d-149	ReLU-239	ReLU-329
BatchNorm3d-60	BatchNorm3d-150	Conv3d-240	Bottleneck-330
ReLU-61	ReLU-151	BatchNorm3d-241	Conv3d-331
Conv3d-62	Bottleneck-152	ReLU-242	BatchNorm3d-332
BatchNorm3d-63	Conv3d-153	Conv3d-243	ReLU-333
ReLU-64	BatchNorm3d-154	BatchNorm3d-244	Conv3d-334
Conv3d-65	ReLU-155	ReLU-245	BatchNorm3d-335
BatchNorm3d-66	Conv3d-156	Bottleneck-246	ReLU-336
ReLU-67	BatchNorm3d-157	Conv3d-247	Conv3d-337
Bottleneck-68	ReLU-158	BatchNorm3d-248	BatchNorm3d-338
Conv3d-69	Conv3d-159	ReLU-249	ReLU-339
BatchNorm3d-70	BatchNorm3d-160	Conv3d-250	Bottleneck-340
ReLU-71	ReLU-161	BatchNorm3d-251	Conv3d-341
Conv3d-72	Bottleneck-162	ReLU-252	BatchNorm3d-342
BatchNorm3d-73	Conv3d-163	Conv3d-253	ReLU-343
ReLU-74	BatchNorm3d-164	BatchNorm3d-254	Conv3d-344
Conv3d-75	ReLU-165	ReLU-255	BatchNorm3d-345
BatchNorm3d-76	Conv3d-166	Bottleneck-256	ReLU-346
ReLU-77	BatchNorm3d-167	Conv3d-257	Conv3d-347
Bottleneck-78	ReLU-168	BatchNorm3d-258	BatchNorm3d-348
Conv3d-79	Conv3d-169	ReLU-259	ReLU-349
BatchNorm3d-80	BatchNorm3d-170	Conv3d-260	Bottleneck-350
ReLU-81	ReLU-171	BatchNorm3d-261	AdaptiveAvgPool3d-351

Conv3d-82	Bottleneck-172	ReLU-262	Conv3d-352
BatchNorm3d-83	AdaptiveAvgPool3d-173	Conv3d-263	BatchNorm3d-353
ReLU-84	Conv3d-174	BatchNorm3d-264	ReLU-354
Conv3d-85	BatchNorm3d-175	Conv3d-265	ResNet-355
BatchNorm3d-86	ReLU-176	BatchNorm3d-266	DataParallel-356
Conv3d-87	ResNet-177	ReLU-267	GRU-357
BatchNorm3d-88	DataParallel-178	Bottleneck-268	BatchNorm1d-358
ReLU-89	Conv3d-179	Conv3d-269	Linear-359
Bottleneck-90	BatchNorm3d-180	BatchNorm3d-270	
Total params: 92835714			
Trainable params: 525570			
Non-trainable params: 92310144			

In spatial encoder module, Tencent Medicalnet 3DResnet model, which was pretrained on 23 medical datasets, is employed to extract spatial features in multi-phase images. The corresponding model structure and pretrained weights are publicly assessable as open-source code (https://github.com/Tencent/MedicalNet).

Appendix E7. Deep learning model training process

In the initial phase of interactive segmentation model training, we focused on training the P-net for 60 epochs. Then, we trained the R-net for 60 epochs based on the best P-net model checkpoint. In both steps, we adopted the stochastic gradient descent optimizer with step learning rate and dice cross entropy loss. During classifier model training, the SETD model was trained with a weighted cross-entropy loss function for 200 epochs. To avoid overfitting, we adopted early stopping based on validation cohort performance. The training process was interrupted when the loss score in validation dataset did not decrease for 30 epochs. To ensure optimal training efficiency and precision, our strategy includes an adaptive adjustment of the learning rate. Specifically, if the validation AUC score does not show any improvement for a consecutive period of 5 epochs, we proactively reduce the learning rate. This adjustment is crucial to prevent the loss function from oscillating around its minimum value, thereby facilitating a more stable and effective convergence during the training process.

Appendix E8. AI-assisted diagnosis case example

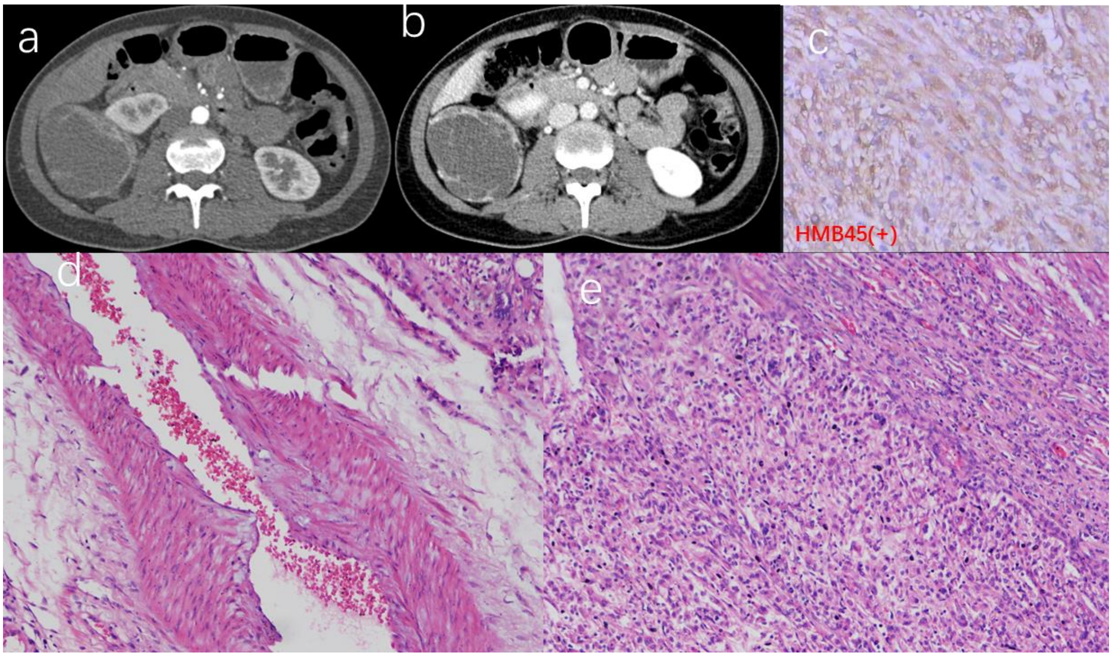

Patient: Female, 37 years old

Imaging: CT scan findings consistent with Bosniak III classification (2019 version)

(a-b) a: corticomedullary phase b: nephrogenic phase

Pathology: angiomyolipoma confirmed (c-e)

CK(-),EMA(-),CD117(-),VIM(+),CK7(-),RCC(-),CD10(-),TLE-3(-),SMA(+),

Desmin(-), S-100(-),SOX-10(-),HMB45(+), MelanA(+),ki67 ＜5%(+)

All pathology images have a 20x pathology magnification.

HMB45 is a common marker to diagnosis the perivascular epithelioid cell tumors

(angiomyolipoma, lymphangioleiomyomatoisis, PEComa, clear cell "sugar" tumor).

The proposed AI system accurately identify it as benign CRLs.

REFERENCES

1 Ronneberger O, Fischer P, Brox T (2015) U-net: Convolutional networks for biomedical image segmentationMedical Image Computing and Computer-Assisted Intervention–MICCAI 2015: 18th International Conference, Munich, Germany, October 5-9, 2015, Proceedings, Part III 18. Springer, pp 234-241

2 Mahootiha M, Qadir HA, Aghayan D, Fretland Å A, von Gohren Edwin B, Balasingham I (2024) Deep learning-assisted survival prognosis in renal cancer: A CT scan-based personalized approach. Heliyon 10:e24374

3 Liu J, Yildirim O, Akin O, Tian Y (2023) AI-Driven Robust Kidney and Renal Mass Segmentation and Classification on 3D CT Images. Bioengineering (Basel) 10

4 Wang G, Zuluaga MA, Li W et al (2019) DeepIGeoS: A Deep Interactive Geodesic Framework for Medical Image Segmentation. IEEE Trans Pattern Anal Mach Intell 41:1559-1572

5 He QH, Tan H, Liao FT et al (2022) Stratification of malignant renal neoplasms from cystic renal lesions using deep learning and radiomics features based on a stacking ensemble CT machine learning algorithm. Front Oncol 12:1028577

6 Gharaibeh M, Alzu’bi D, Abdullah M et al (2022) Radiology Imaging Scans for Early Diagnosis of Kidney Tumors: A Review of Data Analytics-Based Machine Learning and Deep Learning Approaches. 6:29

7 Hsiao C-H, Sun T-L, Lin P-C et al (2022) A deep learning-based precision volume calculation approach for kidney and tumor segmentation on computed tomography images. Computer Methods and Programs in Biomedicine 221:106861

8 van Griethuysen JJM, Fedorov A, Parmar C et al (2017) Computational Radiomics System to Decode the Radiographic Phenotype. Cancer Res 77:e104-e107

Figure E1 legend. Detailed workflow of CT images preprocessing for SETD model training

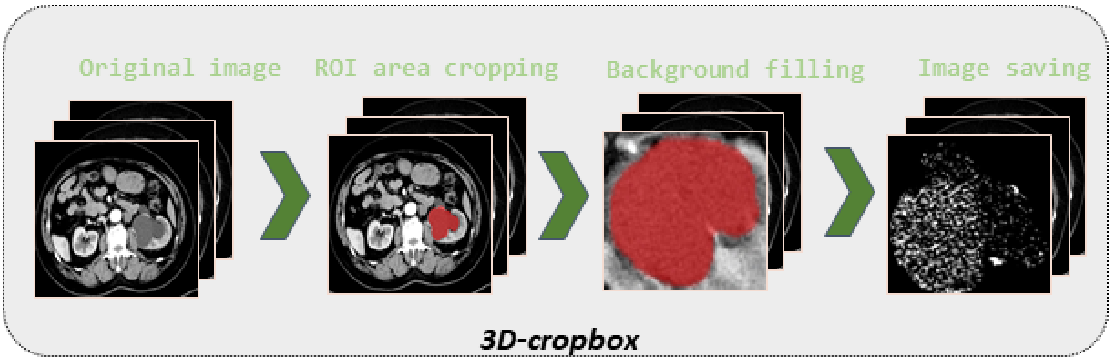

There are three main parts to the 3D-cropbox workflow: cropping of ROI areas, filling of region backgrounds, and adjusting input sizes. The area outside ROI will be filled with black.

Figure E2 legend. Detailed workflow of CT images augmentation for SETD model training

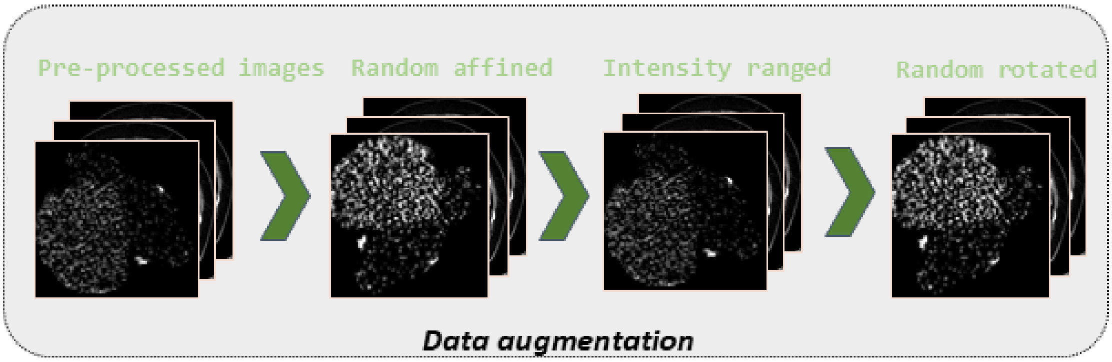

The Random Affine Transformation technique applies an affine transformation to the input images (50% probability). This transformation encompasses random translations, rotations, and scaling operations. Specifically, the random rotation component of this method rotates the input images by a randomly chosen angle within a range of -10.0 to 10.0 degrees, again with a probability of 0.5. Additionally, the Intensity-Ranged method adjusts the pixel values of each image, scaling them to fall within a 0 to 250 range. This method ensures a consistent intensity scale across all processed images.

Figure E3 legend, The confusion matrix for the machine learning model in validation datasets

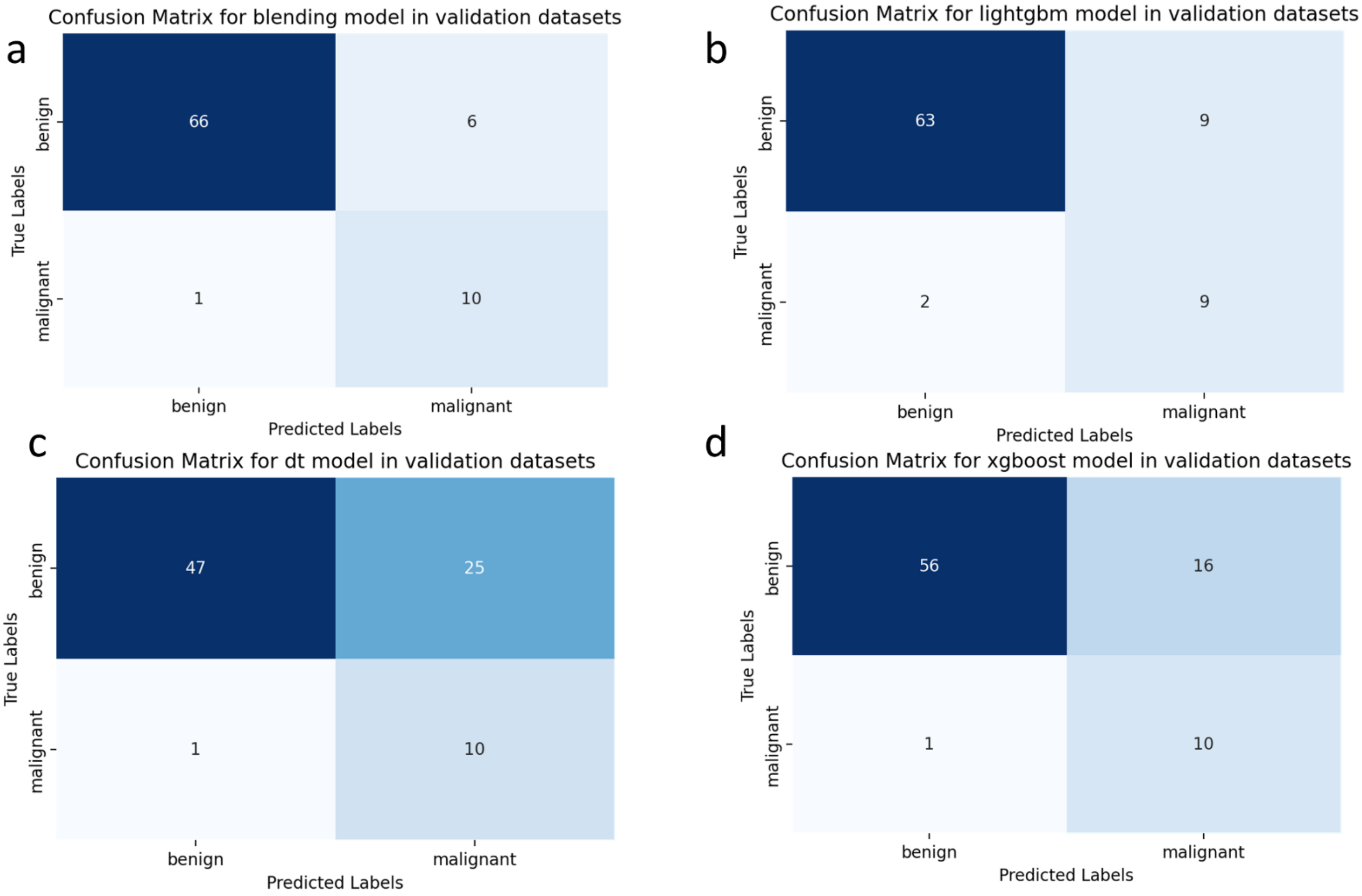

Figure E4 legend. The confusion matrix for the machine learning model in

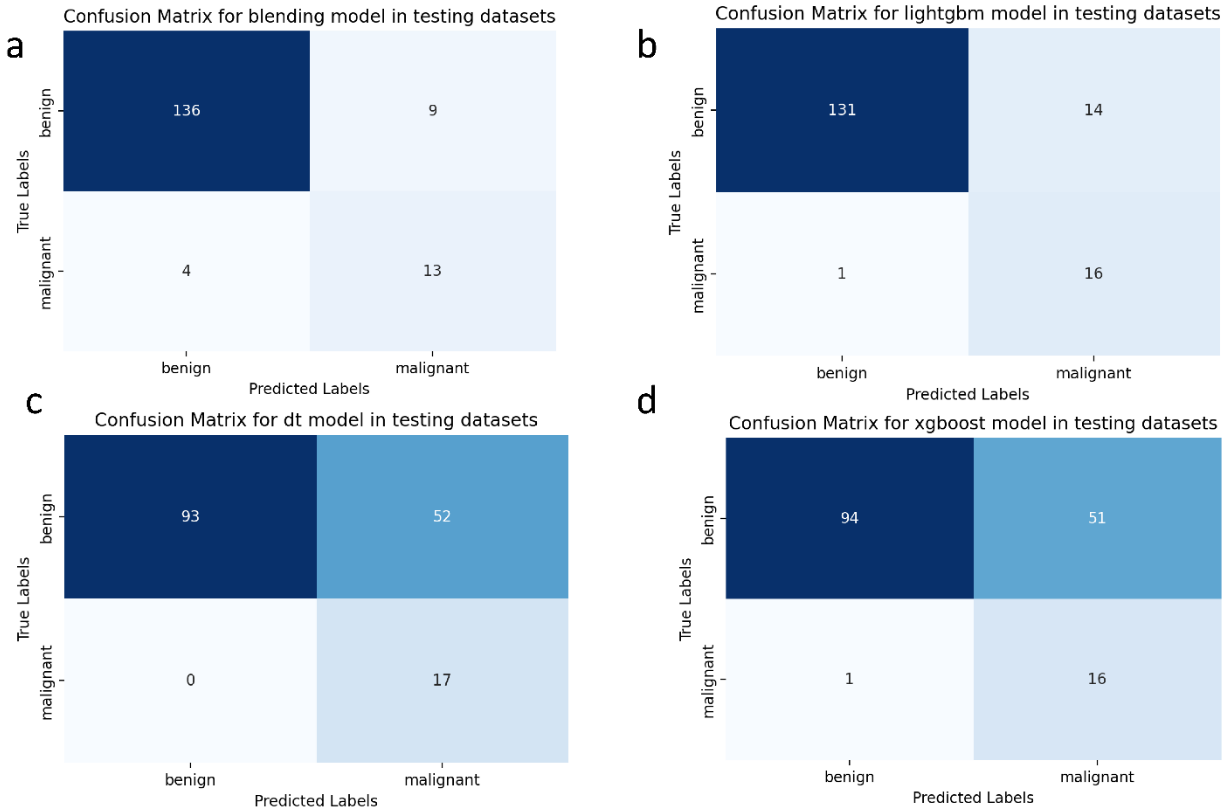

Figure E5 legend. The Calibration curve for the SETD model and machine learning model

a

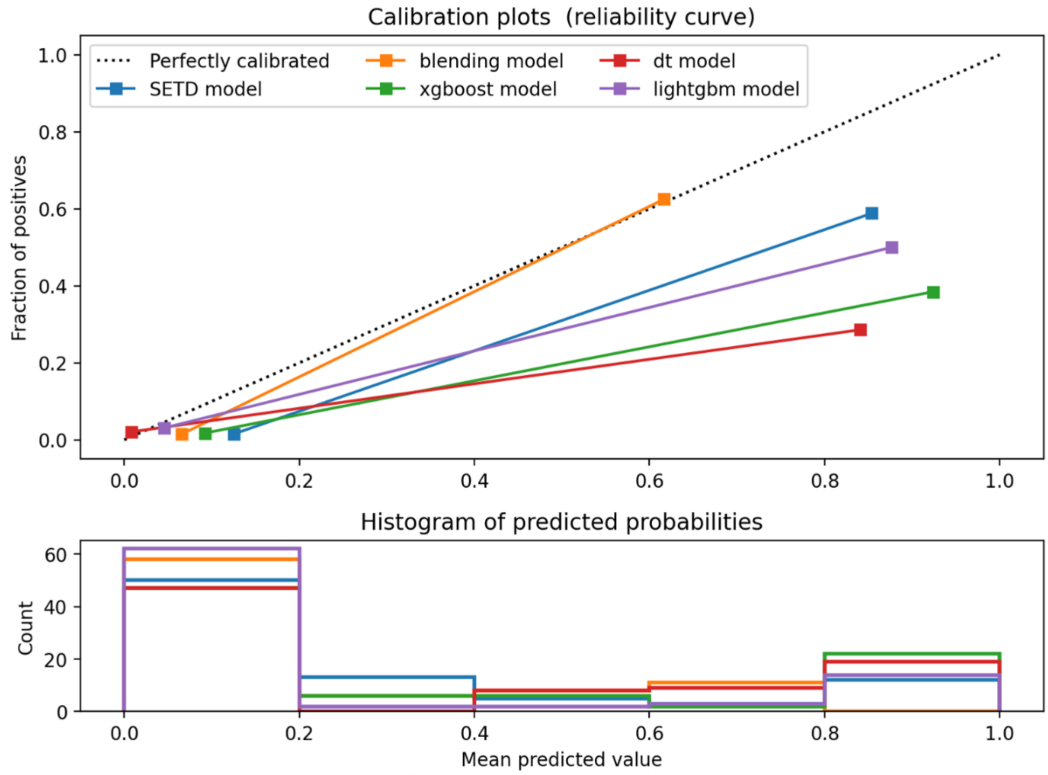

b

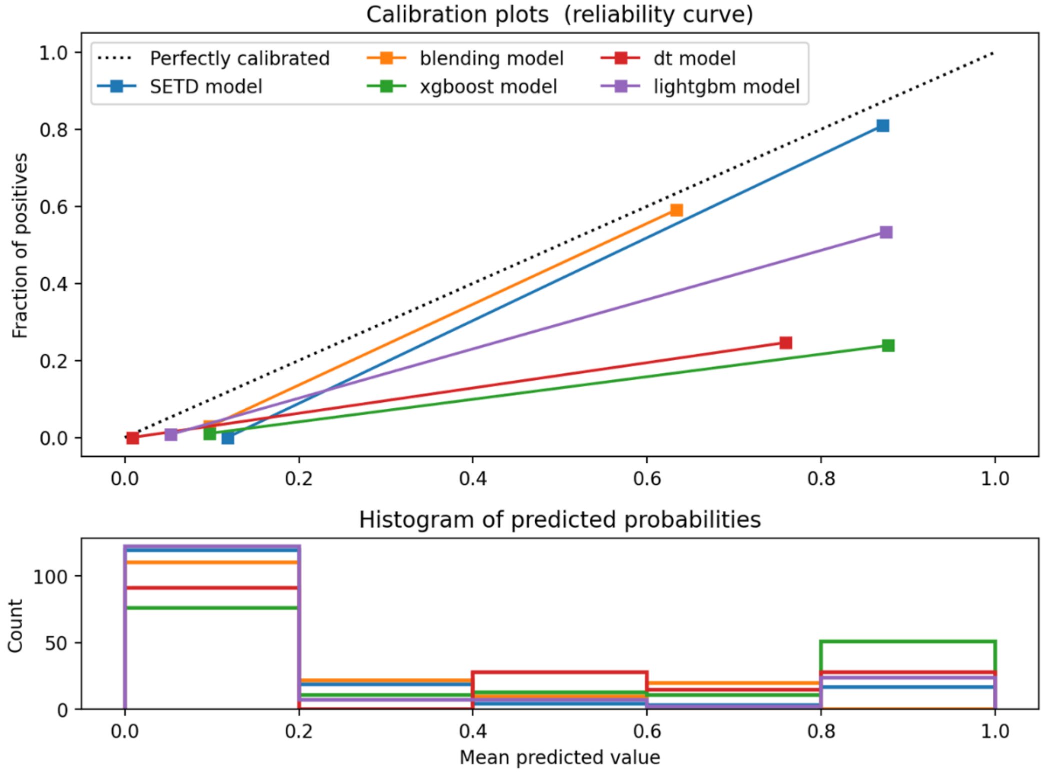

Table E1. Deep learning and radiomics model quality score checklist

Radiomics Quality items	Score
Multi-center study	1
Defined the classification of the model	1
Input from Clinicians for interpretable pipeline development	1
Image protocol quality are well documented and publicly used	2
Description of the hardware used and image reconstruction method	2
Accounted for Preprocessing of the images	1
Imaging at multiple time points	1
Inclusion and exclusion criteria or boundaries of the model defined	1
Use post-processing harmonization to reduce multi-center acquisition variability	1
Cut-off analyses	1
Early stopping method used to avoid the risk of overfitting	1
Comparative analysis between radiomics and deep learning	1
Quality Management System available online with internal audit	4
Adopted bootstrapping resampling method and discrimination plots	2
Adopted bootstrapping resampling method and calibration plots	2
Comparison with previously published radiomics signatures and models	1
Validation is based on three distinct institutes	5
Available detailed evaluation explanations	2
Comparison to 'gold standard'	2
Proved potential clinical utility by decision curve analysis	2
the radiomics model provides the predictions and the clinician’s intervention is required for special (out-of-distribution) cases	4
The algorithm and source code are made publicly available.	1
CT scan protocol, ROI segmentation methods, clinical data are open source	3
Future strategy to update models	1
Total score	43

Table E2. Detailed CT imaging scanning protocols in the training cohort and each validation cohort

Parameter	Training cohort	Validation cohort	External validation cohort1	External validation cohort 2	External validation cohort 3
**CT scanner**	128-slice spiral CT (Siemens) or 64-slice spiral CT (GE)	128-slice spiral CT (GE)	64-slice spiral CT (Toshiba) or 64-slice spiral CT (Siemens)	256-slice scanner (Philips)	128-slice spiral CT (Siemens) or 64-slice spiral CT (GE)
**CT-tube voltage**	100–120 (kV)	90–120 (kV)	100–120 (kV)	100–120 (kV)	100–120 (kV)
**CT-tube current**	125–300(mAs)	125–350(mAs)	125–300(mAs)	125–360(mAs)	125–350(mAs)
**Scanning matrix**	512*512 pixels	512*512 pixels	512*512 pixels	512*512 pixels	512*512 pixels
**Body reconstruction kernel**	yes	yes	yes	yes	yes
**Slice thickness**	1 mm to 7 mm	1 mm to 7 mm	1 mm to 7 mm	1 mm to 7 mm	1 mm to 7 mm
**Contrast volume**	1.5(mL/kg)	1.5(mL/kg)	1.5(mL/kg)	1.5(mL/kg)	1.5(mL/kg)

Table E3. Detailed distribution of Bosniak-2019 classification and pathology results in the SETD model training cohort and external validation cohorts.

pathology analysis& Up to 4 years of imaging follow-up		benign results n=140	malignance results n=55
Training cohort	Bosniak I(n=89)	simple renal cysts(n=89)	(n=0)
	Bosniak II (n=29)	simple renal cysts(n=19)<br>Up to 4 years of imaging follow-up (8)	papillary renal cell carcinoma(n=1)<br>tubulocystic renal cell carcinoma(n=1)
	Bosniak IIF (n=17)	simple renal cysts(n=7)<br>cystic nephroma (n=1)<br>renal angiomyolipoma (n=2)	tubulocystic renal cell carcinoma(n=2)<br>papillary renal cell carcinoma(n=1) clear cell renal cell carcinoma(n=3)<br>multilocular cystic renal neoplasm of low malignant potential (n=1)
	Bosniak III (n=20)	simple renal cysts(n=5)<br>cystic nephroma (n=1)<br>Up to 4 years of imaging follow-up (2)<br>renal angiomyolipoma (n=1)	clear cell renal cell carcinoma(n=6) papillary renal cell carcinoma(n=1)<br>chromophobe renal cell carcinoma(n=1)<br>multilocular cystic renal neoplasm of low malignant potential (n=2)<br>tubulocystic renal cell carcinoma(n=1)
	Bosniak IV (n=40)	renal angiomyolipoma (n=2)<br>cystic nephroma (n=2)<br>Up to 4 years of imaging follow-up (1)	unclassified renal cell carcinoma (n=5), renal collecting duct carcinoma (n=1)<br>clear cell renal cell carcinoma(n=21)<br>papillary renal cell carcinoma(n=5) chromophobe renal cell carcinoma(n=2)<br>multilocular cystic renal neoplasm of low malignant potential (n=1)
**pathology analysis**		**n=72**	**n=11**
validation	Bosniak I(n=35)	simple renal cysts(n=35)	(n=0)
	Bosniak II(n=14)	simple renal cysts(n=14)	(n=0)
	Bosniak IIF	simple renal cysts(n=16)	clear cell renal cell carcinoma(n=2)

**cohort**	(n=20)	mixed epithelial and stromal tumor (1)	multilocular cystic renal neoplasm of low malignant potential (1)
	Bosniak III(n=7)	simple renal cysts(n=5)	clear cell renal cell carcinoma (2)
	Bosniak IV(n=7)	cystic nephroma (1)	clear cell renal cell carcinoma (5)<br>multilocular cystic renal neoplasm of low malignant potential (1)
**pathology analysis**		**n=145**	**n=17**
	Bosniak I (n=104)	simple renal cysts(n=104)	(n=0)
**External**	Bosniak II(n=22)	simple renal cysts(n=20)	multilocular cystic renal neoplasm of low malignant potential (2)
**validation**	Bosniak IIF(n=20)	simple renal cysts(n=17)	multilocular cystic renal neoplasm of low malignant potential (3)
**cohort 1**<br>**cohort 2& cohort 3**	Bosniak III(n=9)	simple renal cysts (n=2)	clear cell renal cell carcinoma(n=3), renal collecting duct carcinoma (n=2)<br>papillary renal cell carcinoma(n=1)<br>multilocular cystic renal neoplasm of low malignant potential (1)
	Bosniak IV(n=7)	simple renal cysts(n=2)	clear cell renal cell carcinoma (3), unclassified renal cell carcinoma (n=2)

CLEAR Checklist v1.0

Note: Use the checklist in conjunction with the main text for clarification of all items.
Yes, details provided; No, details not provided; n/e, not essential; n/a, not applicable; Page, page number

Section	No.	Item	Yes	No	n/a	Page
**Title**						
	1	Relevant title, specifying the radiomic methodology	☑	☐	☐	1
**Abstract**						
	2	Structured summary with relevant information	☑	☐	☐	1
**Keywords**						
	3	Relevant keywords for radiomics	☑	☐	☐	2
**Introduction**						
	4	Scientific or clinical background	☑	☐	☐	2
	5	Rationale for using a radiomic approach	☑	☐	☐	3
	6	Study objective(s)	☑	☐	☐	3-4
**Method**						
Study Design	7	Adherence to guidelines or checklists (e.g., CLEAR checklist)	☑	☐	☐	4
	8	Ethical details (e.g., approval, consent, data protection)	☑	☐	☐	4
	9	Sample size calculation	☐	☐	☑	
	10	Study nature (e.g., retrospective, prospective)	☑	☐	☐	4
	11	Eligibility criteria	☑	☐	☐	4-5
	12	Flowchart for technical pipeline	☑	☐	☐	5
Data	13	Data source (e.g., private, public)	☑	☐	☐	7
	14	Data overlap	☑	☐	☐	5
	15	Data split methodology	☑	☐	☐	5
	16	Imaging protocol (i.e., image acquisition and processing)	☑	☐	☐	5
	17	Definition of non-radiomic predictor variables	☐	☐	☑	
	18	Definition of the reference standard (i.e., outcome variable)	☑	☐	☐	4-5
Segmentation	19	Segmentation strategy	☑	☐	☐	6
	20	Details of operators performing segmentation	☑	☐	☐	5-6
Pre-processing	21	Image pre-processing details	☑	☐	☐	6
	22	Resampling method and its parameters	☑	☐	☐	6
	23	Discretization method and its parameters	☑	☐	☐	6
	24	Image types (e.g., original, filtered, transformed)	☑	☐	☐	6
Feature extraction	25	Feature extraction method	☑	☐	☐	6
	26	Feature classes	☑	☐	☐	6
	27	Number of features	☑	☐	☐	6
	28	Default configuration statement for remaining parameters	☑	☐	☐	6
Data preparation	29	Handling of missing data	☐	☐	☑	
	30	Details of class imbalance	☑	☐	☐	6-7
	31	Details of segmentation reliability analysis	☑	☐	☐	6-7
	32	Feature scaling details (e.g., normalization, standardization)	☑	☐	☐	6-7
	33	Dimension reduction details	☑	☐	☐	6-7
Modeling	34	Algorithm details	☑	☐	☐	7
	35	Training and tuning details	☑	☐	☐	7
	36	Handling of confounders	☑	☐	☐	7
	37	Model selection strategy	☑	☐	☐	7
Evaluation	38	Testing technique (e.g., internal, external)	☑	☐	☐	7
	39	Performance metrics and rationale for choosing	☑	☐	☐	7
	40	Uncertainty evaluation and measures (e.g., confidence intervals)	☑	☐	☐	7
	41	Statistical performance comparison (e.g., DeLong's test)	☑	☐	☐	7
	42	Comparison with non-radiomic and combined methods	☑	☐	☐	7
	43	Interpretability and explainability methods	☐	☐	☑	
**Results**						
	44	Baseline demographic and clinical characteristics	☑	☐	☐	7-8
	45	Flowchart for eligibility criteria	☑	☐	☐	8
	46	Feature statistics (e.g., reproducibility, feature selection)	☑	☐	☐	8
	47	Model performance evaluation	☑	☐	☐	8-9
	48	Comparison with non-radiomic and combined approaches	☑	☐	☐	9
**Discussion**						
	49	Overview of important findings	☑	☐	☐	10
	50	Previous works with differences from the current study	☑	☐	☐	10
	51	Practical implications	☑	☐	☐	11
	52	Strengths and limitations (e.g., bias and generalizability issues)	☑	☐	☐	12

Section	No.	Item	Yes	No	n/a	Page
**Open Science**						
Data availability	53	Sharing images along with segmentation data [n/e]	☐	☐	☑	
	54	Sharing radiomic feature data	☑	☐	☐	7
Code availability	55	Sharing pre-processing scripts or settings	☑	☐	☐	6
	56	Sharing source code for modeling	☑	☐	☐	7
Model availability	57	Sharing final model files	☐	☐	☑	
	58	Sharing a ready-to-use system [n/e]	☑	☐	☐	7

Kocak B, Baessler B, Bakas S, Cuocolo R, Fedorov A, Maier-Hein L, Mercaldo N, Müller H, Orlhac F, Pinto Dos Santos D, Stanzione A, Ugga L, Zwanenburg A. CheckList for EvaluAtion of Radiomics research (CLEAR): a step-by-step reporting guideline for authors and reviewers endorsed by ESR and EuSoMII. Insights Imaging. 2023 May 4;14(1):75. doi: 10.1186/s13244-023-01415-8
